# Safety and Efficacy of Intraventricular Antibiotics for Central Nervous System Infections in Children: A Single-center Observational Study

**DOI:** 10.1097/INF.0000000000005198

**Published:** 2026-02-26

**Authors:** Mona Almarzooqi, Fionnuala Ryan, Seilesh Kadambari, James Hatcher, Dulanka Silva, Sara Boyd, Orlagh McGarrity, Hanna Schmid

**Affiliations:** From the * Department of Paediatric Infectious Diseases, Great Ormond Street Hospital NHS Foundation Trust; † Department of Paediatric Infectious Diseases, St George’s University Hospital NHS Foundation Trust; ‡ Department of Microbiology, Virology and Infection Control, Great Ormond Street Hospital for Children NHS Foundation Trust; § Department of Neurosurgery, Great Ormond Street Hospital NHS Foundation Trust, London; ¶ David Price Evans Global Health and Infectious Disease Research Group, Institute of Systems, Molecular and Integrative Biology, University of Liverpool, Liverpool; ‖ Department of Pharmacy, Great Ormond Street Hospital NHS Foundation Trust, London, United Kingdom.

**Keywords:** intraventricular antibiotics, ventriculoperitoneal, external ventricular device

## Abstract

Intraventricular antibiotics represent a potential adjunct to intravenous antibiotics in central nervous system infections, to achieve adequate drug concentrations in cerebrospinal fluid. In our single-center experience, describing 37 treatment episodes, intraventricular antibiotics mostly targeting shunt-associated central nervous system infections were generally well tolerated and associated with rapid clearance of cerebrospinal fluid. Large randomized controlled trials are needed to better assess treatment efficacy.

The use of intravenous (iv) antimicrobial therapies has various inherent challenges in the treatment of central nervous system (CNS) infections. Their limited ability to penetrate the blood-brain and blood-cerebrospinal fluid (CSF) barrier and thus achieve adequate CSF concentrations, renders the treatment of ventriculoperitoneal (VP) and external ventricular device (EVD) related infections particularly complex.^1^

Intraventricular antimicrobial treatment (IVT) represents a potential adjunctive treatment in these scenarios, by allowing direct delivery of antimicrobials to the site of infection, especially when using an iv compound with limited CSF penetration. IVT has been shown to achieve higher concentrations in the CSF compartment as described in a small randomized controlled trial in adults, although outcomes of bacterial clearance did not differ between iv and IVT routes.^2^ Furthermore, IVT may have a role in treating multidrug-resistant pathogens. However, long-standing safety concerns remain due to findings in a multicenter controlled trial conducted 45 years ago, showing higher mortality rates in infants without indwelling devices treated with additional IVT gentamicin for Gram-negative meningitis.^3^ No further trials in neonates have been completed since to confirm or revoke these findings.^4^

Efficacy data supporting the use of IVT in children with VP/EVD infections is also limited, as is data on tolerability and posology, with, to our knowledge, no randomized controlled trials in children published. A case series, done in adults, suggested IVT may contribute to a quicker decline of CSF white blood cell count (WBC), associated with better functional outcome, hypothesizing that the kinetics of reduction of CSF WBC might rather reflect clinical response than the actual drop in cell count.^5^ More rapid microbiologic clearance, as well as enhanced clinical recovery with shorter durations of both hospital stay and systemic antibiotic treatment, was also observed in another retrospective adult cohort.^6^

We aimed to evaluate current practice at our tertiary pediatric center, where IVT is being administered in combination with systemic antibiotics for shunt or EVD infections, to potentially inform future prospective trials as well as studies on pharmacokinetics and pharmacodynamics.

## MATERIALS AND METHODS

This was a retrospective, single-center observational study, including children ≤18 years treated with IVT at Great Ormond Street Hospital, London, between January 1, 2019 and December 31, 2024, through our electronic medical records system. Data on demographics, clinical, biochemical and microbiologic parameters and antimicrobial treatment were extracted using an anonymized proforma.

Empiric age-adapted dosing for the 2 used IVT agents (vancomycin and gentamicin) was guided by our institution’s internal guideline, extrapolated from the 2017 Infectious Diseases Society of America’s Clinical Practice Guidelines for Healthcare-Associated Ventriculitis and Meningitis. (1) These were adjusted based on trough levels to ensure adequate CSF concentrations at 10 to 20 times the minimum inhibitory concentration of the causative pathogen. CSF trough levels of IVT were obtained every 24 hours, until within range to proceed with the next dose (<15 mg/L for vancomycin and <3 mg/L for gentamicin).

Clinical response was assessed based on clinical and biochemical parameters, including CSF culture, CSF WBC count, 30-day mortality (from administration of first IVT dose), microbiologic failure (defined as ongoing culture positivity in CSF after 72 hours), infection recurrence (defined as culture positivity with same microorganism from CSF, within 30 days after completion of treatment), adverse events and brain imaging pre- and post-IVT.

This project was registered as a service evaluation (number 3751) with the clinical audit/quality service at Great Ormond Street Hospital, in line with the criteria outlined by the National Health Service’s Health Research Authority.

## RESULTS

We identified 37 treatment episodes with IVT in 36 children. The ages ranged from 1 month to 16 years (median 1.75 years), 60% were male (n = 22). Most patients had multiple comorbidities (median = 3), including hydrocephalus with VP shunt or EVD in situ (n = 33/36, 92%), prematurity (n = 13/36, 33%), congenital CNS malformations (n = 8/36, 22%), cardiac (n = 6/36, 17%), respiratory comorbidities (n = 6/36, 17%), CNS tumors (n = 2/36, 6%), kidney disease (n = 2/36, 6%), metabolic (n = 2/36, 6%), endocrine conditions (n = 2/36, 6%) and 1 hematologic malignancy.

Most common treatment indications were VP shunt infection (n = 30/37, 81%), EVD infection (n = 4/37, 11%) and intraventricular reservoir infection (n = 1). Two children were treated for CNS infection not associated with foreign material.

In patients with shunt-associated infections, the most common presenting features were fever (n = 13/37, 35%), vomiting (n = 9/37, 24%) and irritability (n = 6/37, 16%). Other frequently reported symptoms were respiratory distress (n = 5/37, 14%), lethargy (n = 5/37, 14%), swelling or skin changes at the shunt site (n = 6/37, 16%), visible CSF leak (n = 3/37, 8%) and abdominal distension (n = 3/37, 8%). Median CSF WCC was 45, 30 and 8/μL at the beginning, middle and end of the IT antibiotic course, respectively. Median C-reactive protein at presentation was 78 mg/L (interquartile range 151).

Overall, 31 patients (31/37, 84%) had positive CSF cultures, of which 18/31(58%) were still positive at IVT initiation. The most frequently identified pathogens were *Staphylococcus aureus* (10/31), *Staphylococcus epidermidis* (6/31) and *Pseudomonas aeruginosa* (3/31). Further details of CSF culture results are summarized in Table 1. Twenty of 37 (54%) patients received IVT vancomycin (of which 90% were also given vancomycin via iv route) and 17/37 (46%) IVT gentamicin (of which 82% also received an iv aminoglycoside) with a median duration of 11 days for both. The maximum duration for IVT vancomycin was 38 days, and 24 days for gentamicin. All patients received concomitant iv antibiotics.

**TABLE 1.** Biochemical and Microbiological Characteristics of Patients Treated With Intraventricular Antibiotics for Foreign Material Associated Central Nervous System Infections, Divided by Age Group

Total Number of Cases Per Age Group	Indication (Number of Cases)	CSF WCC Before Starting IVT, Range	CSF Culture at Start of IVT (Number of Cases)	IVT Antibiotic and Daily Dose (Number of Cases)	Hours to Negative CSF Culture From Starting IVT (Range)	Possible Toxicity (Number of Cases)
0–3 months						
2	VP shunt infection (1), intraventricular reservoir infection (1)	40–5040	*Streptococcus agalactiae* (1), no growth (1)	Vancomycin 2.5 mg (1) Gentamicin 0.2 mg (1)	0–39	None
>3–6 months						
9	VP shunt infection (7), EVD infection (2)	0–2410	*Enterococcus faecalis* (1), *Enterococcus faecium* (1), *Staphylococcus aureus* (1), *Staphylococcus haemolyticus* (2), *Staphylococcus capitis* (1), *Staphylococcus epidermidis* (1), *Pseudomonas aeruginosa* (1), no growth (2)	Vancomycin 3 mg (7) Gentamicin 0.5 mg (2)	0–365	Respiratory distress (1)
>6 months–2 years						
7	VP shunt infection (7)	0–330	*S. aureus* (1), *S. epidermidis* (5), *S. capitis* (1, coinfection with *S. capitis),* no growth (1)	Vancomycin 5 mg (6) Gentamicin 1 mg (1)	0–55	Vomiting (2), respiratory distress (1)
>2–5 years						
4	VP shunt infection (3), EVD infection (1)	0–3360	*S. aureus* (2), *Streptococcus pyogenes* (1), no growth (1)	Vancomycin 5 mg (2) Gentamicin 2.5 mg (2)	0–20	None
>5–10 years						
5	VP shunt infection (4), EVD infection (1)	91–3680	*P. aeruginosa* (2), *S. aureus* (1), no growth (2)	Gentamicin 2.5 mg (5)	0–55	Increased seizure frequency (1)
>10–18 years						
8	VP shunt infection (8)	2–3600	*S. aureus* (5; 1 MRSA), *E. faecalis* (1), *Cutibacterium acnes* (1), no growth (1)	Vancomycin 5 mg (3) Gentamicin 2.5 mg (5)	0–45	None

CNS indicates central nervous system; CSF, cerebrospinal fluid; EVD, external ventricular drain; iv, intravenous; IVT, intraventricular antimicrobial treatment; MRSA, methicillin-resistant Staphylococcus aureus (methicillin-susceptible where not otherwise stated); VP, ventriculoperitoneal; WCC, white cell count.

Median time to CSF culture negativity was 25 hours, with 17/18 (94%) patients achieving clearance at 72 hours. Median time from first IVT administration to reinsertion of VP shunt was 15 days (interquartile range 12–27; mean 20 ± 11). 2/37 patients (5%) had a culture-positive recurrence of CNS infection within 30 days of stopping IVT: One child presented clinically with a shunt infection, and VP shunt was exchanged for an EVD. The initial CSF culture was positive for *Cutibacterium acnes* with a high WCC (45), and treatment established with IVT vancomycin for 9 days, leading to clinical improvement. CSF culture on the day of VP shunt reinsertion, 1 day after stopping IVT, was again positive for *C. acnes*. This was, however, interpreted as contaminant and treatment was not extended with no unfavorable outcome. The second child was transferred to our hospital from overseas, where he had a recent exchange from VP shunt to EVD due to a previous VP shunt-associated infection with *P. aeruginosa*. Repeat CSF at our institution grew *Enterococcus faecalis* for which intraventricular vancomycin was administered via EVD for 23 days, with negative CSF culture achieved at 52 hours. The CSF culture again isolated *E. faecalis* again at 17 days after stopping IVT. This was managed with iv antibiotics and the patient went on to have a new VP shunt inserted 45 days following the initial start of IVT.

Potential toxicity was recorded in 5 patients. Four of these were showing symptoms of vomiting (n = 2) and respiratory distress (n = 2) while treated with intraventricular vancomycin, and 1 patient who received intraventricular gentamicin experienced increased seizure frequency.

All patients were alive at 30 days; 1 was lost to follow-up after transfer overseas. At 6 months, 1 patient had died due to shunt blockage and deteriorated following complications of increased intracranial pressure, not associated with complications of IVT. One further patient was lost to follow-up after moving back overseas.

Of the 2 children treated for CNS infection not associated with foreign material, 1 had *Escherichia coli* ventriculitis with clinical deterioration despite treatment with iv meropenem. Intraventricular gentamicin was added for 8 days, with clinical improvement and no relapse or recurrence. The second patient had cerebral abscesses in the context of hematologic malignancy, which were positive for *Rothia mucilaginosa* on 16S PCR. After failing to respond to initial iv antibiotics, he received 6 days of intraventricular vancomycin. He clinically improved and had a VP shunt successfully inserted for associated hydrocephalus 45 days after completion of IVT, with no recurrence. Both patients were alive 6 months postreceiving IVT.

## DISCUSSION

In our single-center pediatric experience, IVT mostly targeting shunt-associated CNS infections was generally well tolerated and associated with rapid bacterial clearance of CSF. Our findings were comparable with sterilization rates achieved in a larger retrospective cohort in adults, as well as a smaller descriptive series of 34 children treated with adjunctive IVT through externalized ventricular catheters.^7,8^

Our findings also suggest that intraventricular administration could represent an adjunctive therapy in cases of shunt-associated CNS infections, particularly in patients with limited response to systemic antibiotic therapy or multidrug-resistant infections. Direct comparison with systemic antibiotics alone is lacking, for which to our knowledge, no studies are currently available in the specific pediatric context. There are only limited data published in adults, suggesting more rapid CSF clearance and a drop in WBC, and potentially associated with better clinical outcomes.^4,9,10^ Nevertheless, in the small pediatric series, lack of sterilization of CSF by iv treatment alone was described in a third of their patients, with subsequent bacterial clearance achieved through IVT via EVD.^8^

We describe novel pediatric data in the management of CNS infections using IVT and utilized robust clinical and microbiologic endpoints. However, our study has several limitations, including the lack of a comparator group, retrospective study design and limited interpretation of the tolerability of IVT, including no standardized hearing assessments performed after treatment. We included a small and heterogenous cohort with limited follow-up. We could not observe any increased mortality in our cohort, consistent with the other published small pediatric case series.^8^

Our study provides preliminary data on the potential use of IVT antibiotics in shunt-associated CNS infections, supporting the need for further prospective and comparative data, to better define the optimal dosage, duration and patient selection criteria for this therapeutic approach, to assess its role in treatment and establish monitoring protocols for potential toxicity. This could help assess whether the use of IVT may lead to more rapid sterilization of CSF and hence shorter time to reinsertion of VP-shunts. While randomized controlled trials would provide the highest level of evidence, their feasibility, specifically in this pediatric setting, might be challenged by small case numbers at individual centers and a heterogeneic and often acutely unwell patient cohort, which could be addressed by multicenter collaboration and adaptive trial designs.

## ACKNOWLEDGMENTS

*We thank all involved patients and families, our colleagues in the subspecialities that have led on the care of these children as well as in the antimicrobial stewardship team, Drugs and Therapeutics Committee and Camelia Botnar Microbiology Laboratory.*
